# MANagement of Gallstone disease in the Older patient (MANGO): clinical and quality of life outcomes from a prospective multicentre cohort study

**DOI:** 10.1186/s12893-026-03854-8

**Published:** 2026-06-08

**Authors:** Anna E. Fairclough, Ana D. Gavrila, Charlotte Cossins, Simon K. C. Toh, Alexander R. Darbyshire, Amy Lord, Wessex Research Collaborative, Ayman Darwich, Louise Finch, Atilla Emin, Charlotte Parfitt, Malcolm West, John Primrose, Charles West, Oliver Pickering, Vikash Dodhia, Charlotte Parfitt, Raymond Chaorui Li, Alison Ferguson, Noha Al-Makadma, Samuel McKoy, Orla Busby, Phoebe Carter, Michael Woyton, Vishnu Suresh, Nathan Curtis, Madeleine Taylor, Manish Gupta, Ulysses Hechanova, Thomas Knowles, Aseem Shah, Sarita Agte, Georgina Belt, Stephanie Hannabus, Godwin Dennison, Vimal Mahendran, Nigel Beer, Lucy Pippard, Kate Beesley, Linda Howard, Alison Lewis, Jess Perry, Fenella Welsh, Mark Sidhom, Rikhilroy Patel, Rosin Kelly, Joanne McClintock, Joseph Doyle, Christian Wakefield, Bhavik Patel, Simran Chhugani, Nathan Grundy, Arpita Devashetty, Joanne McClintock, Luca Bonomo, Claire Osey, Chloe Bascombe, Cheryl Lindsay, Javen Ramsami, Suzanne Roffe, Lindsay Rogers, Luca Bonomo, Karen O’Toole, Matthew Bayliss, Charlotte Humphrey, Natasha Tamjidi, Damian Mayo, Alpha Anthony, Luke Bennett, Holly Morgan, Kate Seymour, Fiona Trim

**Affiliations:** 1 Department of General Surgery, Portsmouth Hospitals University NHS Trust, Portsmouth, PO6 3LY England; 2 Department of General Surgery, Dorset County Hospital, Dorchester, DT1 2JY England; 3 Department of General Surgery, Basingstoke and North Hampshire Hospital, Basingstoke, RG24 9NA England; 4 Department of Surgery and Cancer, Imperial College, London, SW7 2AZ England; 5 Department of General Surgery, Southampton General Hospital, 44 Wilton Crescent, Southampton, SO15 7QH England; 6 Department of General Surgery, Stoke Mandeville Hospital, Aylesbury, HP21 8AL England; 7 Department of General Surgery, Yeovil District Hospital, Yeovil, BA21 4AT England; 8 Department of General Surgery, Royal Hampshire County Hospital, Winchester, SO22 5DG England; 9 Department of General Surgery, Royal Bournemouth Hospital, Bournemouth, BH7 7DW England; 10 Department of General Surgery, Poole General Hospital, Poole, BH15 2JB England; 11 Department of General Surgery, Salisbury District Hospital, Salisbury, SP2 8BJ England

**Keywords:** Aged, Aged 70 and over, Cholecystectomy, Cholecystitis, Gallstones

## Abstract

**Background:**

Gallstones commonly cause emergency surgical admission in older adults and are frequently associated with complications. Although cholecystectomy is recommended in the general population, decision-making is complicated by increased comorbidity and frailty in older patients.

**Methods:**

Trainee-led prospective multicentre cohort across nine NHS hospitals. Consecutive emergency admissions in patients aged ≥ 70 years with radiologically confirmed gallstone disease were recruited (November 2022–March 2024). Data were collected at baseline, 30-days and 1-year, including Gastrointestinal Quality of Life Index (GIQLI) scores.

**Results:**

Of 194 patients**,** 36 (18.6%) underwent emergency cholecystectomy, with 158 (81.4%) managed non-operatively at initial presentation. The non-operative group had greater comorbidity burden and frailty. All emergency operations were started laparoscopically (one conversion) with no major complications; median length of stay was similar (7 vs 5 days, *p* = 0.105).

Gallstone-related readmission at 1-year was higher after non-operative management (23.0% vs 2.9%, *p* = 0.024); non-biliary readmissions were similar. One-year mortality was 12.4% vs 0% (*p* = 0.06). Baseline GIQLI was similar. At 30-days, emergency cholecystectomy was associated with the greatest difference in GIQLI score compared to the non-operative group (p ≤ 0.007). At 1-year, GIQLI remained higher after emergency cholecystectomy (123.8 vs 115.6, *p* = 0.039). Forty-three patients had undergone interval cholecystectomy by 1-year.

**Conclusion:**

Emergency cholecystectomy in older patients deemed suitable for surgery is associated with reduced gallstone-related re-admissions at 1-year and higher QoL scores. These findings support consideration of surgery in appropriately selected older patients and further randomised research in this higher risk group.

**Supplementary Information:**

The online version contains supplementary material available at 10.1186/s12893-026-03854-8.

## Introduction

Complications of gallstone disease are a common presentation to the surgical emergency take. Management with emergency or interval laparoscopic cholecystectomy (LC) is the recommended treatment for symptomatic gallstone disease, and it is well proven in the general population that LC is safe, reduces hospital stay and improves quality of life (QoL) [1, 2]. Despite this, the decision to proceed to surgery is not always straightforward; the potential benefits of surgery must be weighed against the age, comorbidities and frailty of the individual patient [3–5].

As our population ages, we are seeing increasing numbers of symptomatic gallstone disease in older patients, for whom surgery is not without significant risk [3, 6, 7]. This leads to higher rates of conservative management in older patients, but may result in increased complications, recurrent symptoms and poor QoL [7–11]. The current evidence base to support clinical decision making for older patients with gallstone diseases comprises only limited, retrospective studies with heterogenous age thresholds to define ‘elderly’. While limited, it is increasingly supportive of intervention in this age group, including emergency or early LC and laparoscopic common bile duct (CBD) exploration [3, 8, 12–14]​. Despite this, in practice we continue to see high rates of conservative management.

The impact of disease and intervention on QoL is crucial to guide decision making in higher risk surgical groups. Currently, there is limited evidence evaluating QoL for gallstone disease in older patients and the resultant cost–benefit of treatments. Some studies present sub-group analysis for QoL by age and have shown benefit of LC in older patients, but generally with a smaller difference than in younger patients, due mostly to a lower baseline QoL [15]. A cost-utility analysis of LC, however, demonstrated a greater benefit of early intervention in older patients, with a larger cost-saving compared to younger groups [16].

Overall, the optimal management of gallstones in the elderly population is still unclear. This study aims to prospectively investigate the management of gallstone diseases in older patients, defined as over 70, and evaluate outcomes of those who undergo emergency (during index admission) or interval LC and those who receive non-operative management.

## Methods

This study was designed and led by the Wessex Research Collaborative and sponsored by Portsmouth Hospitals University NHS Trust. Ethical approval was provided by the Health Research Authority (REC reference: 22/NS/0026) and registered (ISRCTN: 12,668,840). Funding was provided by The Rosetrees Trust and Wessex Clinical Research Network. This study is reported in accordance with the strengthening the reporting of observational studies in epidemiology (STROBE) statement) ​[17].

### Study design and setting

MANGO was a prospective multi-centre cohort study investigating patients, aged 70 years or older, admitted as an emergency with symptomatic gallstone disease during 25/11/2022 to 31/03/2024 (18 months). The study recruited patients from nine NHS hospitals in the southwest of England. The study collected QoL and readmission data at 30 days and 1 year. Follow-up is ongoing with outcomes planned for collection again at 3 years.

### Inclusion criteria

All patients aged 70 years or older, admitted as an emergency with symptoms due gallstone disease, were potentially eligible. To be included in the study patients must have radiological evidence of gallstones on imaging and a confirmed diagnosis, made by a senior clinician, of a gallstone related condition. The eligible diagnoses included were biliary colic, cholecystitis, bile duct stones, cholangitis and acute pancreatitis. Exclusion criteria were patients with prior cholecystectomy or those unable to give informed consent. Recruiting clinicians categorised cholecystitis pragmatically as simple or complicated, and treatment decisions, including emergency cholecystectomy, ERCP, image-guided drainage, or planned interval surgery, were made by the treating teams according to local practice and clinical judgement. The study did not mandate use of the Tokyo Guidelines severity classification.

### Data source

Patients were recruited prospectively by clinical and research teams and baseline data recorded on the index admission. 30-day and 1-year follow-up was performed by telephone, or in person if the patient remained an inpatient. All data were entered prospectively using the secure online database REDCap. Data items collected were patient age, sex, co-morbidities, ASA^1^ grade, clinical frailty score [18]​, diagnosis, radiology findings, factors affecting management decisions and treatment received.

Quality of life was assessed using the Gastro-intestinal Quality of Life Index (GIQLI), a 36-item questionnaire which derives an overall score from several domains: core symptoms, physical, emotional and social dysfunction ​[19]​. A higher score denotes a better QoL. It has been validated as an effective tool in assessing QoL in gallstone disease ​​[20].

### Sample size and bias

The study was powered to detect a difference in readmission rates between the surgery and non-surgery groups at 1 year. We estimated a minimum approximate readmission rate of 30% in patients who do not have surgery compared to 5% in those who do, and that approximately 10% of patients over 70 will have surgery, therefore the enrolment ratio was estimated to be 1:9. Using an alpha of 0.05 and a power of 0.9 the number of patients required was calculated to be 260. We aimed to recruit a minimum of 290 patients to account for a maximum of 10% lost to follow-up.

### Statistical analysis

The study population was divided into patients who underwent emergency cholecystectomy and those managed non-operatively. Baseline characteristics and outcomes were summarised using descriptive statistics, with categorical variables presented as counts and proportions, and continuous variables presented as mean with standard deviation or median with interquartile range, as appropriate. Between-group comparisons were performed using chi-squared tests for categorical variables and rank-based non-parametric tests for continuous variables. Reported group comparisons are unadjusted unless otherwise stated. No imputation was performed for missing GIQLI data, and quality-of-life analyses were conducted as complete-case analyses at each time point. Sensitivity analyses were performed using regression modelling to explore the robustness of the primary comparisons. Data analysis was performed using RStudio, with R version 4.0.0 (R Foundation for Statistical Computing, Vienna, Austria).

## Results

**Fig. 1:**
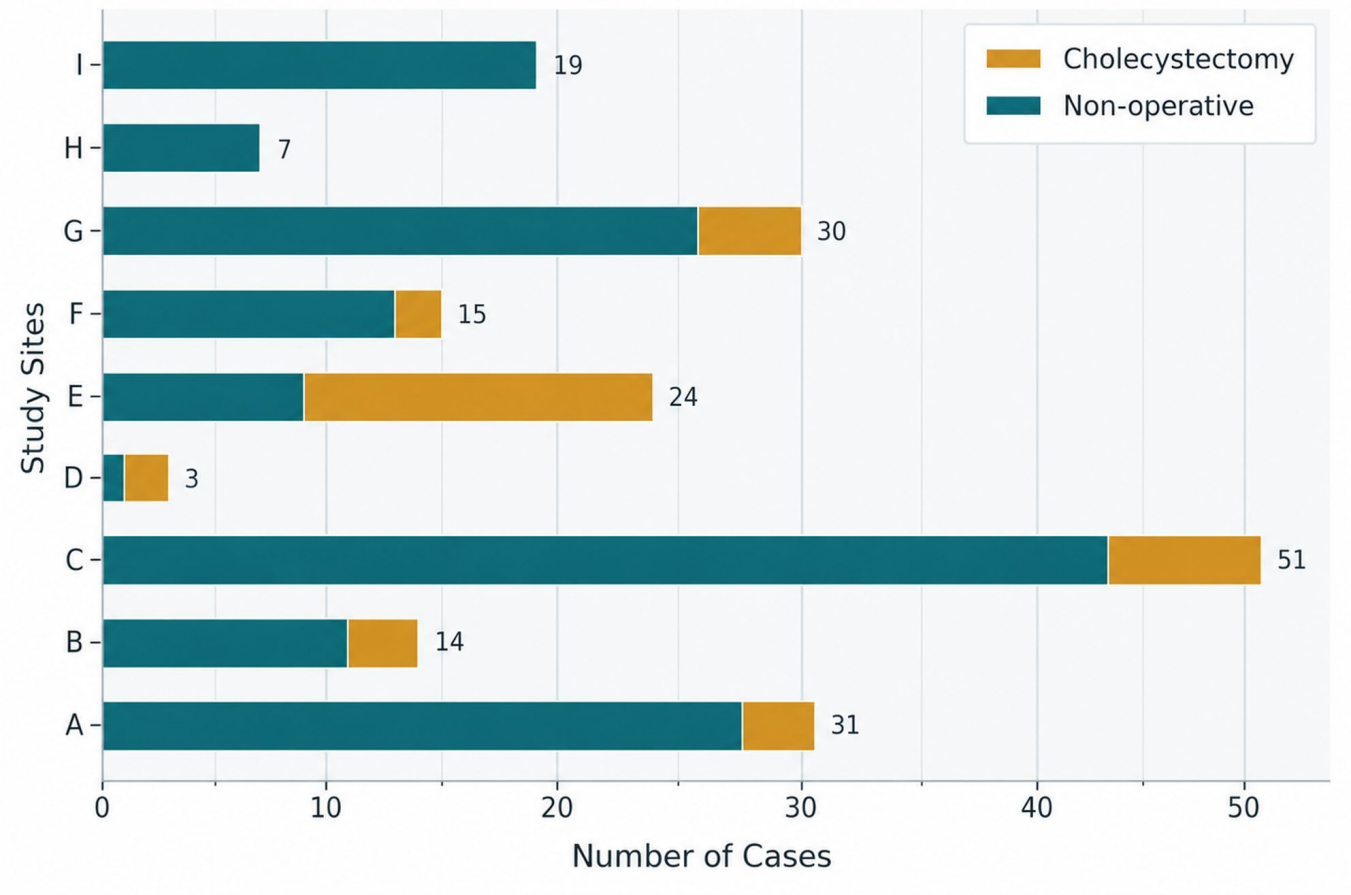
Inpatient management strategy by study site (counts)

**Fig. 2:**
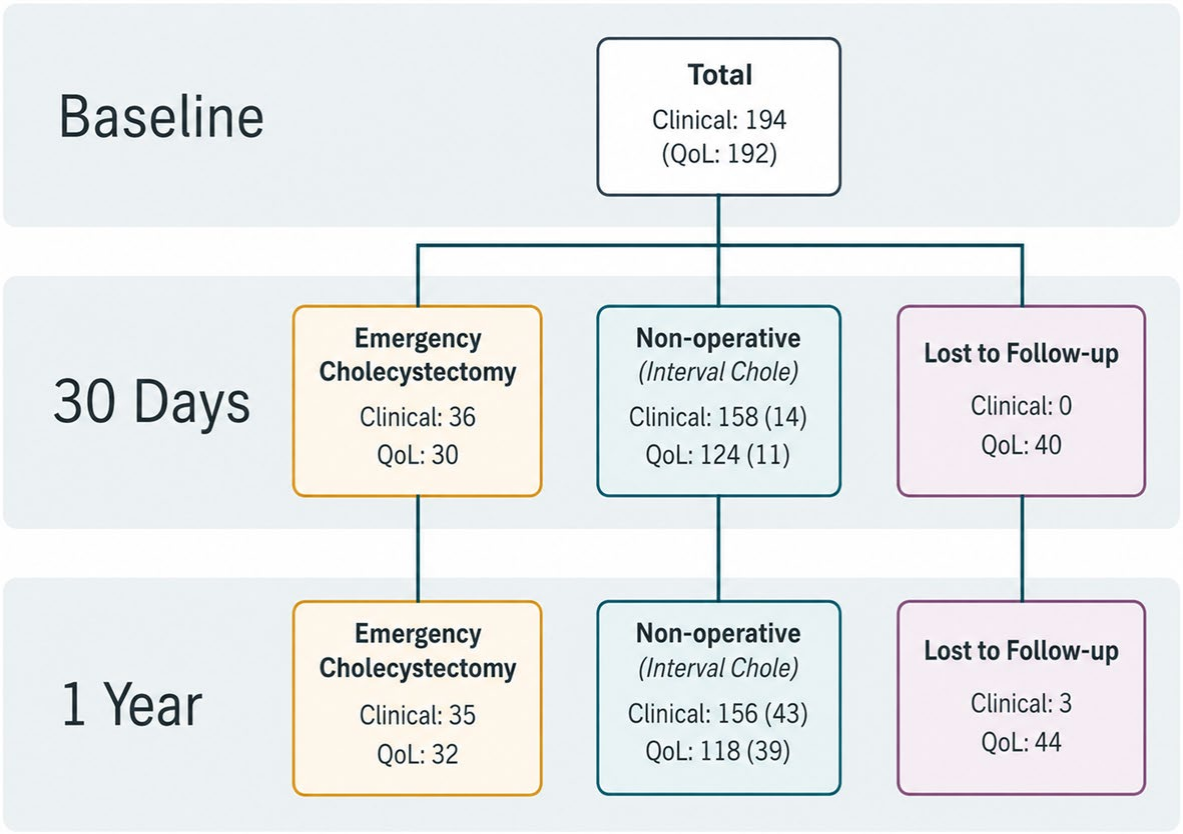
Participant flow chart

A total of 194 patients were recruited from 9 hospitals. The rate of recruitment varied between centres (median 14 [IQR 14–30]) and is displayed in Fig. 1. At 1 year, only 3 patients (1.5%) were lost to follow-up for clinical results. For QoL results, 2 patients did not complete the GIQLI questionnaire at baseline, and loss to follow-up was 40 (20.6%) at 30-days and 44 (22.6%) at 1-year (see Fig. 2).

### Clinical results

**Fig. 3:**
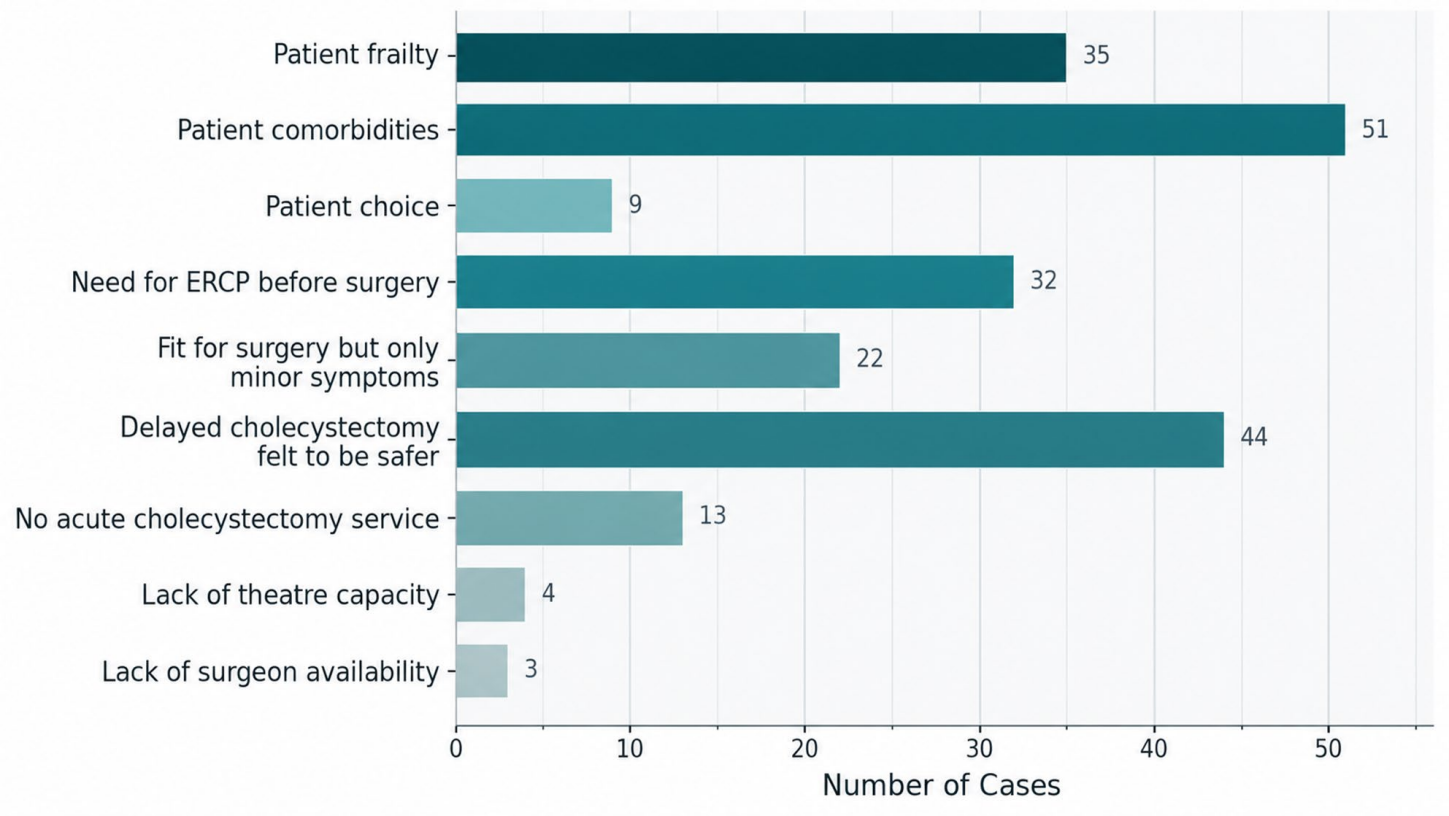
Reasons given for choice of non-operative management (counts) *(Multiple reasons could be selected)*

**Fig. 4:**
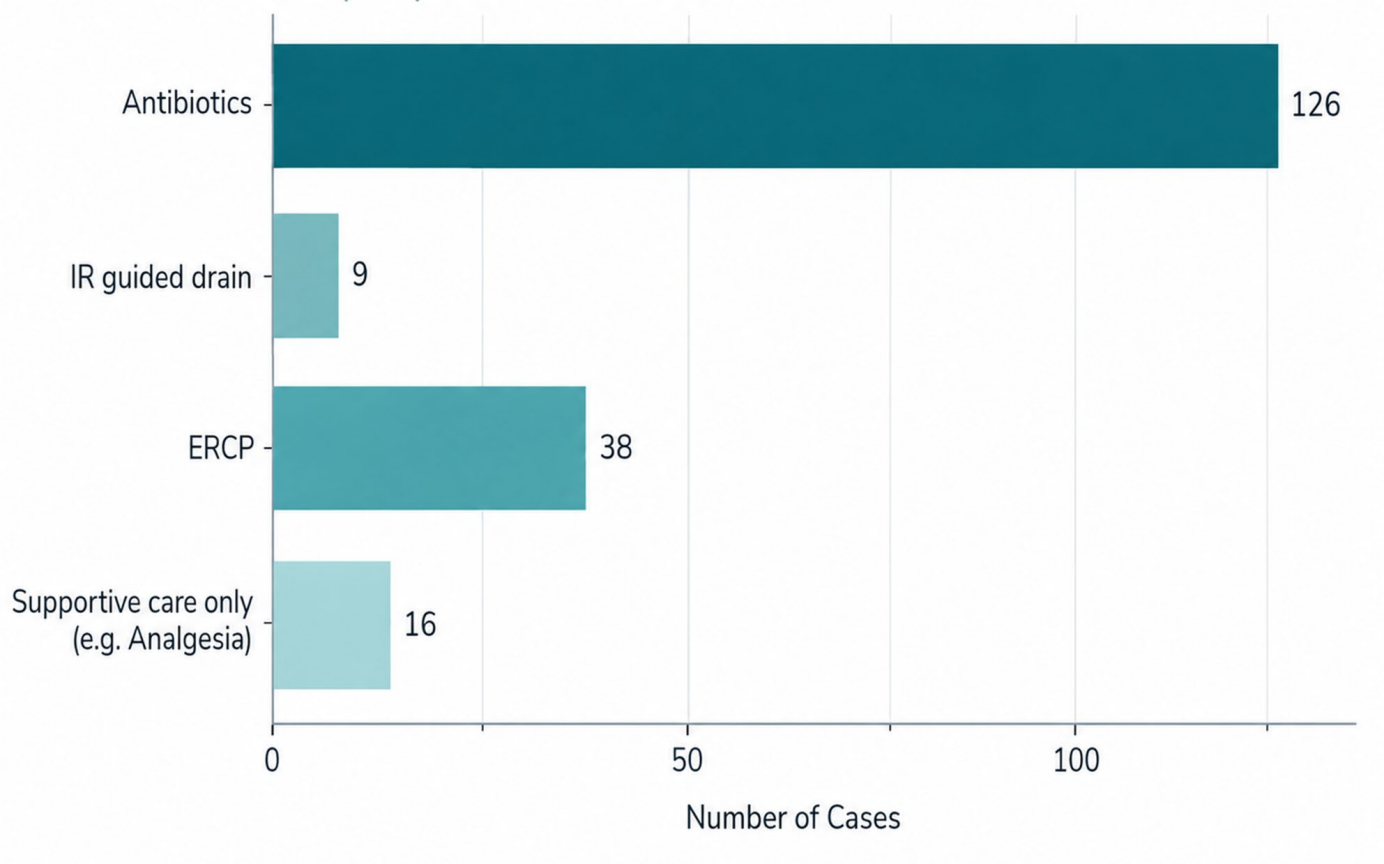
Non-operative management strategy (counts) *Multiple options could be selected*

The majority received non-operative management of their gallstone disease at index admission (81.4%), with 36 patients undergoing emergency cholecystectomy (18.6%). Baseline demographic data analysis is displayed in Table 1, with similar age and sex distributions between groups; however, the study was not powered to establish baseline equivalence. Patient ASA grade, clinical frailty score and rate of major co-morbidities were higher in the non-operative group but did not reach statistical significance in our sample size. Higher rates of cholecystitis were seen in the emergency cholecystectomy group, with biliary colic and bile duct stones more common in the non-operative group. A range of reasons were given for selecting non-operative management and are displayed in Fig. 3, with the most common being patient co-morbidity. Most of the non-operative group received antibiotics, with 38 (24.1%) needing an ERCP and 9 (5.7%) treated with interventional radiology (IR) guided drain (see Fig. 4).

**Table 1:** Patient demographic and diagnostic data based on initial management

	**Emergency Cholecystectomy**	**Non-operative Management**	***p* value**
Total	36 (18.6%)	158 (81.4%)	
Age in years	77 (73—81)	78 (74—83)	0.13
Female Sex	18 (50.0%)	85 (53.8%)	0.82
ASA grade	2 (2—3)	3 (2—3)	0.05
Clinical frailty score			0.08
*None*	30 (88.2%)	100 (69.9%)	
*Mild to Moderate*	4 (11.8%)	38 (26.6%)	
*Severe*	0	5 (3.5%)	
Place of residence			0.01
*Home—independent*	35 (97.2%)	139 (88.0%)	
*Residential home*	1 (2.8%)	0	
*Home—with carers*	0	19 (12.0%)	
Number of major comorbidities	0 (0—1)	1 (0 −1)	0.10
Co-morbidities			
*Heart failure*	5 (13.9%)	30 (19.0%)	0.60
*COPD*	4 (11.1%)	19 (12.0%)	1.00
*Diabetes mellitus*	5 (13.9%)	31 (19.6%)	0.58
*Stroke/TIA*	1 (2.8%)	24 (15.2%)	0.08
*Renal failure*	4 (11.1%)	19 (12.0%)	1.00
*Active malignancy*	0	9 (5.7%)	0.30
Duration of symptoms (hours)	10 (7—15)	7 (2—14)	0.21
Diagnosis			
*Biliary colic*	1 (2.8%)	14 (8.9%)	0.38
*Simple acute cholecystitis*	17 (47.2%)	68 (43.0%)	0.79
*Complicated cholecystitis*	9 (25.0%)	21 (13.3%)	0.13
*Acute pancreatitis*	7 (19.4%)	33 (20.9%)	1.00
*CBD stones with obstructive jaundice*	4 (11.1%)	26 (16.5%)	0.59
*CBD stones with cholangitis*	1 (2.8%)	21 (13.3%)	0.13

Data is presented as either counts (%) or median (IQR)

Of the 36 patients who underwent emergency cholecystectomy, all procedures were commenced using a minimally invasive approach (35 conventional laparoscopic, 1 robot-assisted) with 1 conversion to open surgery and 1 sub-total cholecystectomy. On table cholangiogram was performed in 13 cases (36%) and intra-operative ultrasound in 1 case. Intra-operative bile duct exploration was performed in 4 cases (1 using robotic assisted surgery). Early minor complications occurred in 9 patients. There were 2 reports of post-operative bleeding, 1 respiratory, 3 cardiac problems and 3 acute kidney injuries. There were no major complications, including return to theatre, bile leak, bile duct injury or admission to intensive care.

Patients undergoing emergency cholecystectomy had a slightly longer median length of stay than non-operative management (7 vs 5 days), though this was not statistically significant (see Table 2). Most patients were discharged to their own home without the need for additional support, regardless of the treatment received. At 30-days, there had been 10 unplanned hospital readmissions with gallstone-related disease in the non-operative group, compared to 1 readmission in the operative group (6.3% vs 2.8%) though this did not achieve statistical significance. By 1-year follow-up, there had been 34 emergency readmissions in the non-operative, compared to 1 in the operative group (23% vs 2.9%). Readmission rates due to other health conditions were not significantly different between groups at 30-day and 1-year follow-up (Table 2). 30-day mortality was low, with 3 deaths in the non-operative group, one related to biliary disease, and none in the operative group. However, 1-year mortality was notably numerically higher in the non-operative group (19/156, 12.4%) than in the emergency cholecystectomy group (0/35, 0%), although this did not quite reach statistical significance (*p* = 0.06).

**Table 2:** Initial, 30-day & 1-year outcomes based on initial management

	**Emergency Cholecystectomy**	**Non-operative management**	***p* value**
Initial Admission			
Total	36 (18.6%)	158 (81.4%)	
Mean days of stay (SD)	7.0 (4.8—9.0)	5.0 (3.0—9.0)	0.10
Discharge destination			0.78
*Patient’s own home*	35 (97.2%)	147 (95.5%)	
*Patient’s own home with new need for carers*	1 (2.8%)	5 (3.2%)	
*Rehabilitation hospital*	0	2 (1.3%)	
30-day outcomes			
Total	36 (18.6%)	158 (81.4%)	
Emergency readmission with gallstone related disease	1 (2.8%)	10 (6.5%)	0.65
Emergency readmission with other health condition	5 (13.9%)	20 (13.2%)	1.00
30-day mortality	0	3 (1.9%)	0.93
1-year outcomes			
Total	35 (18.3%)	156 (81.7%)	
Emergency readmission with gallstone related disease	1 (2.9%)	34 (23.0%)	**0.02**
Emergency readmission with other health condition	7 (20.6%)	32 (23.2%)	0.92
1-year mortality	0 (0.0%)	19 (12.4%)	**0.06**

Data presented as count (%) or mean length of stay in days (SD)

Among the 158 patients initially managed non-operatively, 14/158 (8.9%) underwent interval cholecystectomy within 30 days and 43/158 (27.2%) had undergone interval cholecystectomy by 1 year.

### Quality of life results

The quality-of-life results are presented in Table 3 for each follow-up time-point, comparing operative and non-operative groups. The minimally important clinical difference (MCID) for the GIQLI is reported as 4–11 points in patients undergoing elective laparoscopic cholecystectomy for symptomatic gallstones [15, 21].

**Table 3:** Quality of life scores for baseline, 30-day and 1-year follow up

	Emergency Cholecystectomy	Non-operative Management	p value
Baseline			
Patients	35 (18.2%)	157 (81.8%)	
Symptoms score	53.4 (11.4)	51.1 (10.8)	0.26
Emotional score	11.1 (4.8)	10.7 (4.9)	0.63
Physical score	12.7 (6.3)	11.6 (5.8)	0.32
Social score	13.2 (4.4)	13.1 (3.9)	0.93
Total score	90.4 (21.4)	86.5 (20.9)	0.32
30-day Follow-up			
Patients	30 (19.5%)	124 (80.5%)	
Symptoms score	67.2 (7.1)	61.0 (9.9)	0.001
Emotional score	17.5 (2.4)	13.8 (4.7)	0.001
Physical score	19.8 (4.9)	16.5 (6.2)	0.007
Social score	16.8 (3.0)	14.3 (4.0)	0.002
Total score	121.3 (13.1)	105.5 (20.0)	0.001
1-year Follow-up			
Patients	32 (21.3%)	118 (78.7%)	
Symptoms score	67.5 (7.5)	63.8 (10.8)	0.08
Emotional score	17.6 (3.1)	16.3 (4.2)	0.11
Physical score	20.6 (5.0)	18.4 (5.9)	0.05
Social score	18.2 (2.4)	17.6 (3.0)	0.24
Total score	123.8 (14.4)	115.6 (21.1)	0.04

Data presented as counts (%) or mean (SD)

Missing GIQLI data were low at baseline (2/194, 1.0%), with loss to QoL follow-up of 40/194 (20.6%) at 30 days and 44/194 (22.6%) at 1 year. At 30 days, baseline characteristics were similar between participants with and without available QoL data, a sensitivity analysis is shown in Supplementary Table 1. At 1-year, missing QoL data were more common in older, frailer patients with higher ASA grade and greater care needs but were not significantly associated with initial management group.

At baseline, QoL scores were similar between groups. At 30-day follow-up, participants who had undergone emergency cholecystectomy had higher total GIQLI scores than those who received non-operative management (121.3 vs 105.5, *p* = 0.001), with a between-group difference exceeding the published MCID range. At 1-year, total GIQLI remained higher in the emergency cholecystectomy group than in the non-operative group (123.8 vs 115.6, *p* = 0.04), with a between-group difference that remained within the published MCID range. This difference persisted despite the 11 and 39 patients who had undergone interval cholecystectomy by 30-day and 1-year follow-up in the non-operative group. Supplementary Table 2 shows the QoL scores for the non-operative group with interval cholecystectomy included and excluded; with no notable difference.

## Discussion

This prospective cohort study presents the 30-day and 1-year outcomes of emergency admissions due to gallstone disease in patients over 70. Reflective of current practice, fewer patients underwent emergency surgery during their index presentation of gallstone disease. For those who received emergency surgery, there were fewer readmissions for gallstone disease and an improved QoL both at 30-days and 1-year, consistent with other studies [3, 7, 10, 11, 22]. The significant difference in QoL between the two groups is an important finding, pivotal for management decisions. The observation that 1-year QoL was similar for interval cholecystectomy patients and to the rest of the non-operative group needs to be considered, adding to existing evidence [23, 24]. Overall, our findings add prospective observational evidence that, in selected older patients, emergency cholecystectomy is associated with favourable clinical and QoL outcomes compared with initial non-operative management; however, these findings are subject to confounding by indication and should not be interpreted as demonstrating treatment superiority.

The 1-year mortality in the non-operative group was notably numerically higher than in the operative group (12.4% vs 0%, *p* = 0.06). This difference likely reflects baseline differences in frailty, comorbidity, and selection for surgery. Without case matching or a randomised control, we cannot conclude any link to treatment choice in our cohort. The non-operative group had a re-admission rate of 23% at 1 year, which suggests that current non-operative strategies are effective for many patients, particularly those not considered suitable for surgery. This is in keeping with current evidence suggesting favourable outcomes with emergency LC, while also recognising a role for non-operative strategies in selected patients. In gallstone pancreatitis, ERCP alone is protective against recurrence but appears inferior to LC, with one study reporting an 11% biliary recurrence rate [22, 24, 25].

Several limitations of our study must be acknowledged. Study recruitment did not meet protocol targets due to slower than expected recruitment and was powered to detect a difference in readmission rates at 1 year rather than QoL. However, as a trainee-led, low-budget project, this highlights the real-world challenges of conducting multicentre studies without dedicated research infrastructure. The sample size restricts our ability to draw conclusions on rare but critical outcomes, such as bile leak, bile duct injury and return to theatre. In addition, the inclusion of several distinct gallstone-related diagnoses introduces clinical heterogeneity, and the study was not sufficiently powered to support reliable subgroup analyses by presenting diagnosis.

Our cohort also under-represents patients with cognitive impairment or advanced frailty. The exclusion of patients unable to provide informed consent likely resulted in under-representation of patients with dementia, nursing-home residence, and advanced frailty. As these groups commonly present with gallstone-related emergencies, our cohort may represent a relatively healthier subset of older patients, which limits generalisability. This reflects the recognised difficulties in recruiting from this group due to consent and ethical challenges.

Although informative, our results do not definitively identify the optimal management strategy for emergency presentations of gallstone disease in the elderly. Rather, it highlights that older patients who are felt to be suitable for emergency laparoscopic cholecystectomy may have improved outcomes because of this. This study provides key evidence to justify further large-scale and stratified research.

## Conclusions

In summary, patients selected for emergency cholecystectomy had fewer gallstone-related readmissions and higher QoL scores than those initially managed non-operatively. Mortality was numerically higher in the non-operative group, likely reflecting greater frailty, comorbidity, and treatment-selection factors. By 1-year follow-up around half of recruited patients had undergone emergency or interval cholecystectomy, suggesting that operative management can be feasible in selected older patients. Patients who underwent interval cholecystectomy had similar 1-year QoL to those managed non-operatively. These findings provide prospective observational data to inform decision-making and support the need for larger, stratified studies in older patients with gallstone disease.

## Supplementary Information

Supplementary Material 1.

Supplementary Material 2.

## Data Availability

The datasets generated and analysed for this study are not publicly available due to participant confidentiality and institutional data governance policies. Data can be made available from the corresponding author on reasonable request. Data are stored in a secure REDCap (Research Electronic Data Capture) database hosted by the study institution.

## Abbreviations

ASA: American Society of Anesthesiologists (ASA) Physical Status Classification System
CBD: Common bile duct
ERCP: Endoscopic Retrograde Cholangiopancreatography
GIQLI: Gastrointestinal Quality of Life Index
IQR: Interquartile range
IR: Interventional Radiology
LC: Laparoscopic cholecystectomy
QoL: Quality of Life
SD: Standard deviation
