# Supplementary material for: MANagement of Gallstone disease in the Older patient (MANGO): clinical and quality of life outcomes from a prospective multicentre cohort study
Source: BMC Surg. 2026 Jun 8;26:532. doi: 10.1186/s12893-026-03854-8 (PMC13465662; doi:10.1186/s12893-026-03854-8)
Supplement: Supplementary file 1 [file 12893_2026_3854_MOESM1_ESM.docx]

***Supplementary Table 1***: Baseline characteristics for participants with missing QoL data vs. those with QoL data at Initial, 30-day & 1-year follow-up

|  | **Missing QoL** | **QoL Present** | **p value** |
| --- | --- | --- | --- |
| **Initial Admission** | | | |
| **Total (%)** | 2 (1.0) | 192 (99.0) |  |
| **Age (median (IQR))** | 87.0 (83.5 to 90.5) | 78.0 (74.0 to 82.2) | 0.133 |
| **Sex** |  |  |  |
| Female | 2 (100.0) | 101 (52.6) | 0.533 |
| Male | 0 | 91 (47.4) |  |
| **ASA (median (IQR))** | 3.0 (3.0 to 3.0) | 3.0 (2.0 to 3.0) | 0.232 |
| **Frailty** |  |  |  |
| *None* | 1 (50.0) | 129 (73.7) | 0.671 |
| *Mild to Moderate* | 1 (50.0) | 41 (23.4) |  |
| *Severe* | 0 (0.0) | 5 (2.9) |  |
| **Place of Residence** |  |  |  |
| *Home - independent* | 1 (50.0) | 173 (90.1) | 0.157 |
| *Home – with carers* | 1 (50.0) | 18 (9.4) |  |
| *Residential home* | 0 | 1 (0.5) |  |
| **Duration of Symptoms** | 8.0 (4.5 to 11.5) | 8.5 (2.8 to 15.0) | 0.783 |
| **Inpatient Management** |  |  |  |
| *Cholecystectomy* | 1 (50.0) | 35 (18.2) | 0.814 |
| *Non-operative* | 1 (50.0) | 157 (81.8) |  |
| **30-day follow-up** | | | |
| **Total (%)** | 40 (20.6) | 154 (79.4) |  |
| **Age (median (IQR))** | 78.0 (72.0 - 81.2) | 78.0 (74.0 - 83.0) | 0.762 |
| **Sex** |  |  |  |
| Female | 18 (45.0) | 85 (55.2) | 0.33 |
| Male | 22 (55.0) | 69 (44.8) |  |
| **ASA (median (IQR))** | 3 (2.0 - 3.0) | 2.5 (2.0 - 3.0) | 0.138 |
| **Frailty** |  |  |  |
| *None* | 26 (65.0) | 104 (75.9) | 0.333 |
| *Mild to Moderate* | 13 (32.5) | 29 (21.2) |  |
| *Severe* | 1 (2.5) | 4 (2.9) |  |
| **Place of Residence** |  |  |  |
| *Home - independent* | 37 (92.5) | 137 (89.0) | 0.75 |
| *Home – with carers* | 3 (7.5) | 16 (10.4) |  |
| *Residential home* |  | 1 (0.6) |  |
| **Duration of Symptoms** | 10.0 (3.5 to 13.5) | 7.5 (2.2 to 15.0) | 0.766 |
| **Inpatient Management** |  |  |  |
| *Cholecystectomy* | 6 (15.0) | 30 (19.5) | 0.674 |
| *Non-operative* | 34 (85.0) | 124 (80.5) |  |
| **1-year follow-up** | | | |
| **Total (%)** | 41 (21.5) | 150 (78.5) |  |
| **Age (median (IQR))** | 81.0 (78.0 to 85.0) | 77.0 (73.0 to 81.0) | <0.001 |
| **Sex** |  |  |  |
| Female | 22 (53.7) | 79 (52.7) | 1 |
| Male | 19 (46.3) | 71 (47.3) |  |
| **ASA (median (IQR))** | 3.0 (2.8 to 3.0) | 2.0 (2.0 to 3.0) | <0.001 |
| **Frailty** |  |  |  |
| *None* | 18 (45.0) | 109 (81.3) | <0.001 |
| *Mild to Moderate* | 18 (45.0) | 24 (17.9) |  |
| *Severe* | 4 (10.0) | 1 (0.7) |  |
| **Place of Residence** |  |  |  |
| *Home - independent* | 31 (75.6) | 140 (93.3) | 0.002 |
| *Home – with carers* | 10 (24.4) | 9 (6.0) |  |
| *Residential home* | 0 | 1 (0.7) |  |
| **Duration of Symptoms** | 7.0 (1.0 to 13.0) | 10.0 (5.0 to 15.0) | 0.095 |
| **Planned Inpatient Management** |  |  |  |
| *Cholecystectomy* | 3 (7.3) | 32 (21.3) | 0.068 |
| *Non-operative* | 38 (92.7) | 118 (78.7) |  |
