# Supplementary material for: MANagement of Gallstone disease in the Older patient (MANGO): clinical and quality of life outcomes from a prospective multicentre cohort study
Source: BMC Surg. 2026 Jun 8;26:532. doi: 10.1186/s12893-026-03854-8 (PMC13465662; doi:10.1186/s12893-026-03854-8)
Supplement: Supplementary file 2 [file 12893_2026_3854_MOESM2_ESM.docx]

***Supplementary Table 2***: QoL data for non-operative management group including ad excluding interval cholecystectomy.

*Data presented as counts (%) or mean (SD).*

|  | **Non-operative management**  **(inc. interval cholecystectomy)** | **Non-operative management**  **(exc. interval cholecystectomy)** |
| --- | --- | --- |
| **30-day Follow-up** | | |
| **Patients** | **124 (80.5%)** | **113 (73.4%)** |
| *Symptoms score* | *61.0 (9.9)* | *61.1 (10.0)* |
| *Emotional score* | *13.8 (4.7)* | *13.8 (4.8)* |
| *Physical score* | *16.5 (6.2)* | *16.3 (6.2)* |
| *Social score* | *14.3 (4.0)* | *14.1 (4.0)* |
| ***Total score*** | ***105.5 (20.0)*** | ***105.4 (20.1)*** |
| **1-year Follow-up** | | |
| **Patients** | **118 (78.7%)** | **79 (52.7%)** |
| *Symptoms score* | *63.8 (10.8)* | *63.6 (10.8)* |
| *Emotional score* | *16.3 (4.2)* | *16.3 (3.9)* |
| *Physical score* | *18.4 (5.9)* | *18.0 (5.9)* |
| *Social score* | *17.6 (3.0)* | *17.6 (2.7)* |
| ***Total score*** | ***115.6 (21.1)*** | ***115.1 (20.5)*** |
